# Medical Affairs in Pharmaceutical Companies and Related Pharmaceutical Regulations in Japan

**DOI:** 10.3389/fmed.2021.672095

**Published:** 2021-08-09

**Authors:** Hideki Maeda

**Affiliations:** Department of Regulatory Science, Faculty of Pharmaceutical Sciences, Meiji Pharmaceutical University, Kiyose, Japan

**Keywords:** medical affairs, medical science liaison, Japan, pharmaceutical company, clinical trials

## Abstract

Medical affairs has received a lot of attention in recent years in Japan, but it is also often misunderstood or poorly understood in the healthcare industry in Japan. In the United States, the function of medical affairs has been established for a long time, whereas its history in Japan is relatively short. Many scandals in clinical trials occurred with inappropriate relationship between medical doctors and the sales departments of pharmaceutical companies from 2012. These incidents undermined confidence in clinical trials in Japan and triggered the enforced separation of sales departments from the conduct of post-marketing clinical trials and evidence generation. There have been growing compliance issues identified in marketing and sales practices, and off-label promotion is also becoming an issue in Japan. These issues resulted in the establishment of independent medical affairs departments from sales departments in pharmaceutical companies operating in Japan. Due to the short history of medical affairs in Japan, the roles and responsibilities vary between companies in Japan. Medical affairs departments aim to fulfill unmet medical needs through the generation of scientific evidence and to deliver scientific value to key stakeholders and patients. People working in medical affairs need to engage in scientific exchange activities with key opinion leaders independent of sales departments. Through these activities, medical affairs ensures that patients receive optimal medical care. Medical affairs in Japan is still developing, and its roles, responsibilities, and functions are improving. This article covers the history of medical affairs in Japan and the current status and future perspectives of medical affairs in Japan.

## Introduction

Medical affairs (MA) is a function receiving increasing attention in Japanese pharmaceutical companies in recent years. In foreign pharmaceutical companies, MA has a long history of more than 50 years since its establishment (1). In contrast, MA in Japan has a short history and there was no such department in Japanese pharmaceutical companies until 2005. The closest department of MA in a Japanese pharmaceutical company was called Academic Information department, which was affiliated with the sales or marketing departments before 2005. The Diovan scandal, a misconduct case related to a post-marketing clinical trial of an antihypertensive agent, valsartan, in 2012, was the trigger for the establishment of departments charged with creating and building post-marketing clinical data and evidence independent of sales departments in Japanese pharmaceutical companies (2, 3). There have been growing compliance issues identified in marketing and sales practices (4) and off-label promotion is also becoming an issue in Japan (5). As a result, MA departments, independent of sales departments, have become common among Japanese pharmaceutical companies. Although the concept of MA has started to permeate more widely in Japan, healthcare professionals (HCPs), medical students, and pharmacy students, as well as employees in the pharmaceutical industry, are still sometimes unaware of MA. Recognition of MA in Japan is not yet high, and the concept has not yet been fully established. From the perspective of proper use of drugs, it is important for all stakeholders to be aware of MA.

The main purpose of MA activities is to build scientific and medical evidence to meet unmet medical needs (potential demands for unmet medical care) and to conduct activities to disseminate the results to HCPs. MA departments also need to collaborate with business activities, utilize advanced and up-to-date scientific knowledge, and conduct scientific exchange with external experts. By fulfilling these roles, the ultimate goal is to provide optimal medical care to patients. MA has recently undergone rapid development, but it is still developing and continues to evolve in Japan.

This article addresses MA as a priority in pharmaceutical companies, providing a review of the current situation and history of MA in Japan. Summaries of MA in Japan have not been well-disseminated in the English language. Directions along which MA should continue to develop and provide some perspectives on future directions for MA in Japan are also discussed.

## History of MA

### Global History of MA

MA, while a new function in Japan, has been present in the United States for a relatively long time (6, 7). Upjohn (Pfizer) launched a medical science liaison (MSL) team in 1967, with the role of providing medical and scientific education for sales representatives in charge of building relationships with Key Opinion Leaders (KOLs). Since around 2000, issues related to the promotion of off-label uses have become much more common, and the Office of Inspector General (OIG) compliance program was implemented in the United States (8). That process prompted the separation of MA and sales departments with respect to donations and clinical trial implementation, and a movement emerged to separate MSL functions from sales representatives in the United States. In Europe, the MSL functions became independent of sales departments in response to such developments in the United States, and MA departments have become important in Europe and America. MSL functions are also present in Asia (9), Russia (10), and Africa (11), becoming popular functions in pharmaceutical companies.

### History of MA in Japan

**Figure 1:**
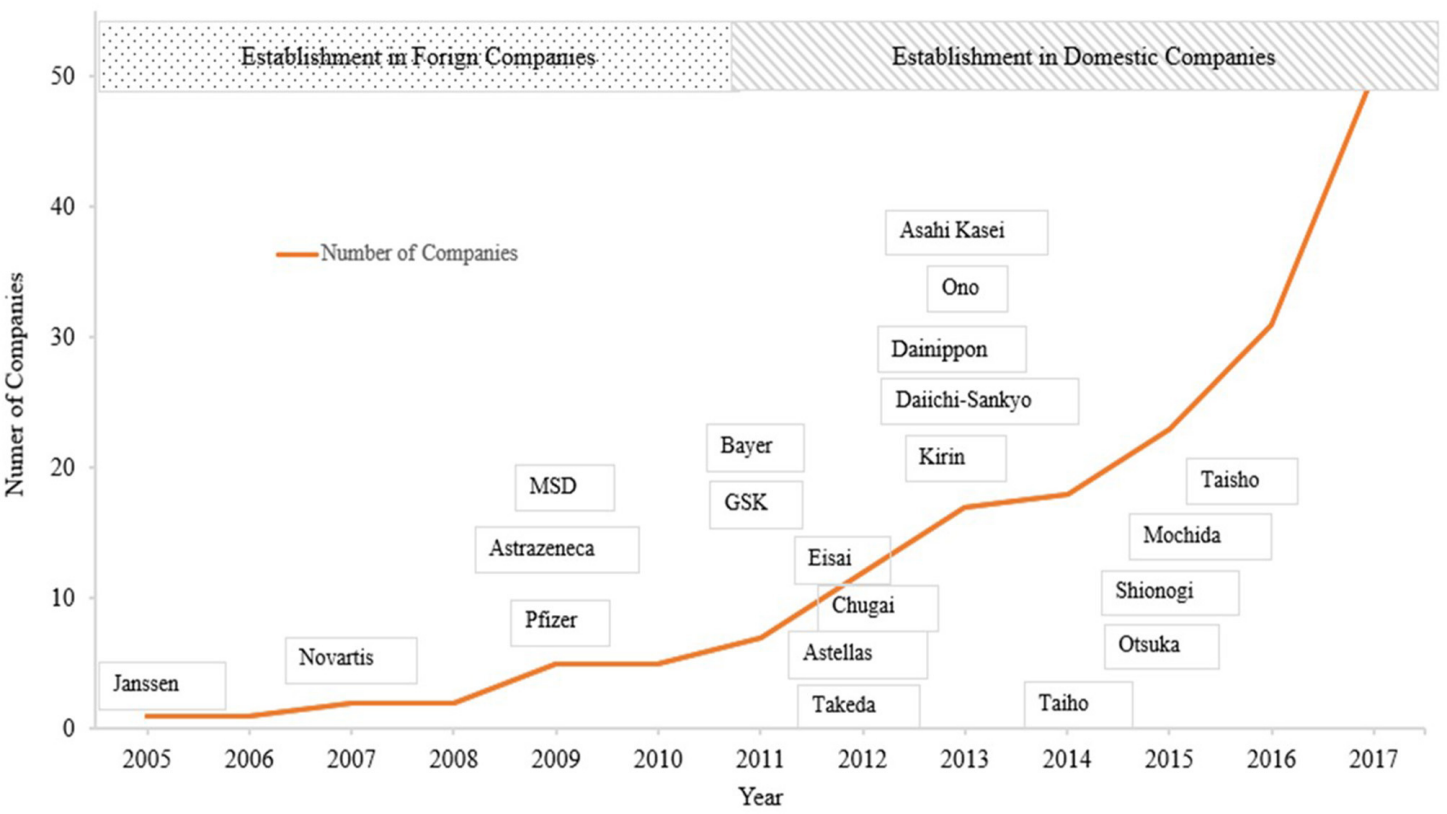
Establishment of MA departments for pharmaceutical companies in Japan.

Table 1 shows major events related to MA from the first establishment of MA functions abroad to the latest issues, mainly in Japan. Around 2005, establishment of MA departments started among foreign-affiliated companies in Japan. In 2005, Janssen Pharmaceuticals launched an MA department, followed by similar developments in foreign companies (12). For more than 5 years, domestic companies lagged behind foreign companies, importing their concepts and ideas from foreign companies or from overseas, and introducing them in a manner tracking the evolution of foreign companies. From 2012, domestic companies started to establish MA departments. Since then, many companies have established MA departments, and more than 80% of pharmaceutical companies are currently considered to have such departments (12) (Figure 1).

**Table 1:** Major Events Related to Medical Affairs in Japan.

**Year**	**Events**
1967	Medical Science Liaison etablished in Upjohn US
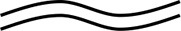
2005	Foreign companies started establishment of Medical Affairs department in Japan (Janssen, Novartis, MSD, AstraZeneca, Pfizer)
2012	Diovan incidents and other misconduct incidents related to post-marketing clinical trials
2012	Domestic companies started establishent of Medical Affairs department in Japan (Takeda, Astellas, Chugai, Eisai)
2013 -	Medical Affairs department continue to be established in a succession of domestic and foreign companies
2014	Pharmaceutical and Medical Device Regulatory Science Society of Japan (PMRJ) established the Regulatory Science Expert Certification System as a method of certifying medical affairs personnel
	Japanese Association of Pharmaceutical Medicine started Medical Science Liaison accreditation system by third-party
2015	European Pharmaceutical Federation (EFPIA) Japan issued the position of Medical Science Liaison and the activity guideline
2016	Pharmaceutical Research and Manufacturers of America (PhRMA) announces Guiding Principles for Medicl Science Liaisons
2017	EFPIA Japan publishes guidelines on Medical Science Liaisons
2018	Clinical Trials Act (Act No. 16 of April 14, 2017) implemented
	Pharmaceutical and Medical Device Regulatory Science Society of Japan (PMRJ) made recommendations and statement on medical affairs
2019	Japan Pharmaceutical Manufacturers Association (JPMA) announce consensus statement on Medical Affairs activities
	Guideline for Sales Information Provision Activities Support for Prescription Drugs implemented

When embarking on MA or MSL activities, most companies to date have either hired mid-career employees or transferred employees from other departments within the company. In recent years, however, several companies (Eisai, Shionogi, Novartis, Bayer, etc.) have been hiring new graduates with a doctorate or Master's degree.

### Movement of Pharmaceutical Organizations and Societies

In 2015, the Japanese Association of Pharmaceutical Medicine (JAPhMed) held the first symposium on MA, followed by a symposium by the Pharmaceutical and Medical Device Regulatory Science Society of Japan (PMRJ) (13) and a symposium on MA at the Drug Information Association (DIA) Japan (14) and other organizations, leading to the formation of communities across companies. Through such activities, discussions have been held regarding the roles of MA, Key Performance Indexes (KPIs), behaviors, and appropriate interactions with sales departments, and momentum has been growing to devise unified standards for MA in the pharmaceutical industry. At present, the ideas, methods, and standards that have arisen in each company are being standardized.

Furthermore, especially for MSLs, higher scientific degrees and certifications of scientific and medical knowledge have become necessary to ensure adequate exchanges of scientific knowledge with medical professionals and KOLs, and to meet the global standards. Accreditation and certification systems in MA have also been started on this basis. First, in July 2014, the PMRJ established the Regulatory Science Expert Certification System as a method of certifying MA personnel (15). In October 2014, a third-party MSL certification system was initiated by JAPhMed (16, 17). The PMRJ certification is a system to qualify individuals, while the JAPhMed accreditation is a unique system to certificate organizations. As of December 2020, a total of four pharmaceutical companies, including AstraZeneca and Takeda, had been accredited by the JAPhMed (14).

### The Clinical Trials Act and Investigator-Initiated Trials in Japan

The Diovan incident in 2012 revealed falsification of data in large-scale clinical trials conducted under investigator-initiated trials after marketing, and all associated papers (18, 19), including papers in related research, were retracted (20). As a result of that incident, confidence in clinical research from Japan plummeted worldwide. Since then, industry, regulatory authorities, and academia have unified to restore confidence in investigator-initiated trials. Efforts have been made to ensure the reliability of clinical trials, implement research systems, and address the legislation underpinning them, and to develop measures to prevent any recurrence of such devastating problems. In December 2014, the “Ethical Guidelines for Medical and Health Research Involving Human Subjects (21)” were revised, and monitoring and auditing were also required for investigator-initiated trials, with consideration to ensuring reliability and scientific quality. The Clinical Trials Act (22) was implemented in April 2018, requiring investigator-initiated trials to comply with laws ensuring the reliability of clinical trials.

## Current Status of MA (Positioning and Roles)

In this section, current status of members, organizational structures, and the roles and responsibilities of MA in Japan are summarized.

### Circumstances Surrounding MA in Japan

A decline has recently been seen in the number of medical representatives (MRs; sales representatives) in Japan. The number of MRs peaked at 65,752 in 2013 and has since declined by 500 to 1,000 annually, reaching 57,158 in 2019 (23). Against this backdrop, MA functions have been reinforced with the establishment of MA departments in Japan. In particular, from the perspective of securing employment, some situations have arisen in which sales alumni have been transferred to MA departments (12).

### Organizational Structure of MA in Japan

The organizational structure of MA departments in Japan appears partially attributable to this transfer of sales personnel to MA departments. In the early days, MA departments in some companies were still affiliated with the sales department. However, in light of lessons learned from the Diovan incident, very few companies currently seem to have MA and sales functions within the same department. As shown below, the organizational structure of MA seems to have shifted into three types:

1) Research and Development (R&D) type: The MA department is under the umbrella of the R&D department and is mainly engaged in medical activities such as medical planning and evidence generation.
2) Independent type: The MA department exists as an independent department in the headquarters and conducts wide-ranging medical activities, including identification of medical needs, creation of medical plans, generation of evidence, and dissemination of information.
3) Coexistent type: MA activities are conducted by both a business unit (therapeutic area) with MA personnel and an independent MA department. MA personnel in the business unit are responsible for planning and implementing medical activities for each product, while the independent MA department reports directly to the company president and is responsible for planning division-type operations such as the supervision of medical activities and donation management.

### Roles and Responsibilities of MA and MSLs in Japan

The operations of the MA department are diverse, and the roles and responsibility of each company are quite variable in Japan, but the tasks that MA generally undertake can be classified into the following four categories:

- Identification of unmet medical needs from the medical field by MSLs who are field-based MA experts engaged in scientific discourse with KOLs;
- Development of medical plans in the post-marketing strategy for maximizing the value of each drug and optimizing the appropriate use of drugs;
- Planning and implementation of post-marketing clinical trials and surveillance, database studies and pharmaco-epidemiological studies with real-world data to accumulate evidence based on medical plans, and support of investigator-initiated clinical trials; and
- Dissemination of scientific and neutral information to KOLs/HCPs and response to inquiries from HCPs *via* call centers and scientific booths at conferences.

In the above, the main roles and responsibilities of MSLs include “identification of unmet medical needs from medical field” and “scientific exchange with KOLs.” In addition, the MSL is also responsible for planning and implementing advisory boards, and other field activities. Because of the variety of MA tasks as described above, many companies divide MA into several groups or departments according to the specific needs of the company (24, 25). Recently, MA in some companies has played a role as a contact point for patient organizations and in advancing the field of Health Technology Assessment (HTA), as well as in compliance functions for sales information provisions. In addition, some pharmaceutical companies are also responsible for external services such as academic societies, scholarships, and donations. These activities are not limited to medical personnel. Recently, more and more companies have been concentrating their functions in order to allow comprehensive responses to external demands such as environmental regulations, compliance, and patient organizations. MA is expected to play an increasingly important role as above in the future.

## Guideline for Sales Information Provision

### Background to the Guideline for Sales Information Provision Activities

On September 25, 2018, the Guideline for Sales Information Provision Activities Support for Prescription Drugs (26) was issued, with the aim of eliminating improper marketing promotion activities undertaken by pharmaceutical companies, such as exaggeration of the effectiveness of drugs or concealment of safety concerns. Subsequently, a first Q&A (27) was issued on February 20, 2019, a second Q&A (28) was issued on March 29, 2019, and a third Q&A (29) was issued on September 6, 2019, by the Ministry of Health, Labour and Welfare in Japan (MHLW). These guidelines and Q&As have stipulated the principles for the provision of sales information by pharmaceutical companies and have also stipulated that a supervisory department (as the department responsible for monitoring sales information provision activities and determining whether the materials used are appropriate) be established in pharmaceutical companies. The MHLW hopes to ensure the proper use of prescription drugs and thereby improve health and hygiene. These guidelines are not only applicable to MRs, but all employees of pharmaceutical companies (including MSLs) must comply with these guidelines.

### Response to the Guidelines for Sales Information Provision Activities of MA

In October 2015, the Corporate Ethics Committee of the Japanese branch of the European Pharmaceutical Federation (EFPIA) issued a position on MSL and the activity guideline (30). In addition, in February 2016, the guideline of the Pharmaceutical Research and Manufacturers of America (PhRMA) (31) was issued, another guideline from the EFPIA was issued in October 2017 (32), and the PMRJ then made recommendations on MA (33). Lastly, the Japan Pharmaceutical Industry Association (Pharmaceutical Industry Association) compiled a “Basic Concept” document on MSL activities in April 2019 (34). On the other hand, concerns have been raised that inappropriate information provision activities are being carried out by MSLs, and the MHLW has been conducting undercover investigations at hospitals nationwide since to crack down on activities by the MRs and MSLs of pharmaceutical companies (35). According to various investigations, inappropriate provision of sales information was reported for 39 drugs in 2016, 52 drugs in 2017, and 64 drugs in 2018 (35). This is still a work in progress, but the issue can be resolved if MA and sales departments are separated more firmly, with the sales department conducting appropriate information–provision activities, and the MA department providing scientific information.

## Discussion

In this section, issues related to the current MA in Japan, future directions, and future prospects are discussed.

### Independence of MA Functions

Many personnel in MA departments in Japan come from the sales department. This is very different from the situation in the United States and Europe, where most members of MA departments are medical doctors or PhD. This is a key characteristic in the Japanese MA situation. Considering the origin of the personnel, a change in the mindset of members is necessary in Japanese MA. For that, the most major pharmaceutical companies in Japan use a “washout” period of training, where the sales team mind is “washed” together with robust scientific training. In addition, clear standards and lines of demarcation need to be set between MA and sales roles, and avoiding duplication of work is also important. Red lines must be established separating sales and MSL teams. Some companies forbid joint medical visits where potential “off-label” questions could appear. The only allowed interactions are reserved for administrative and/or informal connections. As described above, MA functions must be conducted independent of sales functions. It is equally important to ensure appropriate coordination between MA and sales functions in terms of company-level strategy.

### Evaluation of MA

Unlike the situation in sales departments, numerical evaluation of MA department employees is difficult. For MA in Japan, where many employees still come from sales, evaluation methods need to be considered and developed to maintain and improve the motivation of employees. Discussions on KPIs for MA are currently underway within the industry, and it is important to consider indicators related to KPIs within the industry beyond companies. The cooperation of HCPs is also essential. It would be necessary to incorporate evaluation of MSL performance by physicians into KPIs of MSLs. However, physicians including KOLs must view the MSL team as a scientific partner, focused on science and not on sales. MSLs cannot be “super sales teams,” allowed to sell, covered by a “science.”

### Human Resources in MA

In specific therapeutic areas, MA personnel need to have the ability to exchange high-level scientific information with top-level experts. Personnel with high ability and scientific backgrounds thus need to be secured and trained. Working for a pharmaceutical company is still not considered a major career pathway for medical doctors in Japan. In addition, students in faculties of Pharmacy have changed from a 4-year to a 6-year education system. As a result, pharmacy students only have a bachelor's degree on graduation. In the MA field, where higher degrees are required, pharmacy graduates need to upgrade their own educational background while working for companies. In addition, conducting skill improvement, training, and human resource development would be hard to conduct for individual companies, and industry-wide efforts are necessary.

### Others

In pharmaceutical companies, MA departments are the only departments that can generate post-marketing data. In addition, the generation of evidence through clinical research is one of the major roles of MA. MA should promote clinical trials from scientific and neutral standpoints. Taking to heart the lessons learned from the Diovan incident, MA can promote investigator-initiated trials for the true benefit of patients. However, it is important to realize that investigator-initiated trials must “always” come from investigators, not bushed by pharmaceutical companies. In addition, a crucial role of MA is to bridge the gap between the R&D departments of pharmaceutical companies and external KOLs and researchers. Collaboration with other regions and globally is also important. Through the above activities, MA departments are best placed to earn the trust of stakeholders such as patients, HCPs, and regulatory authorities, and can thus be considered the core source of earned trust in pharmaceutical companies.

## Conclusion

MA departments are best placed to earn the trust of stakeholders such as patients, HCPs, and regulatory authorities, and can thus be considered the core source of earned trust in pharmaceutical companies.

## Author Contributions

The author confirms being the sole contributor of this work and approved it for publication.

## Conflict of Interest

The author declares that the research was conducted in the absence of any commercial or financial relationships that could be construed as a potential conflict of interest.

## Publisher's Note

All claims expressed in this article are solely those of the authors and do not necessarily represent those of their affiliated organizations, or those of the publisher, the editors and the reviewers. Any product that may be evaluated in this article, or claim that may be made by its manufacturer, is not guaranteed or endorsed by the publisher.
